# Combined effects of handgrip strength and sensory impairment on the prevalence of cognitive impairment among older adults in Korea

**DOI:** 10.1038/s41598-022-10635-9

**Published:** 2022-04-25

**Authors:** JuHee Lee, Yujin Suh, Jungah Park, Go-Un Kim, Sumi Lee

**Affiliations:** 1 Mo-Im Kim Nursing Research Institute, Yonsei Evidence Based Nursing Centre of Korea: A JBI Affiliated Group, College of Nursing, Yonsei University, Seoul, 03722 Korea; 2 College of Nursing, Health Science & Human Ecology, Dong-Eui University, Busan, 47227 Korea; 3 College of Nursing, CHA University, Pocheon, 11160 Korea; 4 College of Nursing, Inje University, Busan, 47392 Korea; 5 Department of Nursing, Graduate School, Yonsei University, Seoul, 03722 Korea

**Keywords:** Health care, Medical research

## Abstract

Older adults commonly experience concurrent lower handgrip strength and sensory impairment. However, previous studies have analyzed the individual effects of either handgrip strength or sensory impairment on cognitive impairment. To address this gap, this study investigated the combined effects of handgrip strength and sensory impairment on cognitive impairment among older adults. In total, 2930 participants aged 65 and older were analyzed using 2014–2018 data from the Korean Longitudinal Study of Aging. Participants underwent assessments of handgrip strength (grip dynamometer), sensory impairment (self-reported responses), and cognitive impairment (Korean version of the Mini-Mental State Examination). Low handgrip strength, compared to normal handgrip strength, was associated with cognitive impairment. In participants with low handgrip strength, vision and hearing impairment were associated with cognitive impairment (odds ratio [OR] 1.36, 95% confidence interval [CI] 1.06–1.75; OR 2.58, 95% CI 1.77–3.78, respectively) compared to those with normal handgrip strength. Participants with low handgrip strength and dual sensory impairment had the highest OR for cognitive impairment (OR 3.73, 95% CI 2.65–5.25). Due to the strong association of low handgrip strength and dual sensory impairment with cognitive impairment, people living with low handgrip strength and dual sensory impairment should be classified as a high-risk group for cognitive impairment and should be prioritized for interventions.

## Introduction

Cognitive function in older adults is an important health-related factor for successful aging while maintaining independence in daily life in the community. Impaired cognitive function causes problems with memory, performance, judgment, and language abilities^1^. It severely affects individuals’ activities of daily living, such as eating, dressing, and going to the toilet^2^. Partial or full dependence on family and caregivers increases older adults’ risk of frequent healthcare utilization, institutionalization, hospitalization, dementia, reduced quality of life, and death^3^.

Recent research on cognitive impairment has focused on its association with handgrip strength (HGS)^4,5^. HGS is often used as a surrogate of the total power from the upper limb muscles. HGS is known to exhibit differences according to age, sex, and body mass index (BMI)^6,7^. Some degree of variation in HGS is observed depending on nutritional status, physical activity level, inflammation, and disease^8^. Low HGS has been associated with old age^9,10^. Significant correlations were found between low HGS and cognitive function in older adults^11,12^ and were related to mobility, balance, performance and memory impairment^9^. Conversely, high HGS was reported to be a protective factor against cognitive decline^10^. A recent study identified a bidirectional relationship between HGS and cognitive function^13^. In addition, a longitudinal study verified the significance of the association between HGS and cognitive function over time^5^.

Sensory impairment related to aging is often overlooked because sensory problems are common in older adults. Nonetheless, researchers have begun to pay attention to the relationship between sensory impairment and cognitive function^14^. When stimuli are received through sensory receptors in organs such as the eyes, ears, nose, tongue and skin, nerve impulses are transmitted to the central nervous system^15^. However, in the process of aging, sensory reception is reduced, or problems arise due to functional impairment of the sensory organs, resulting in decreased stimulation of the cranial nerves^16^. Sensory impairment in older adults affects homeostasis, static equilibrium, and perceptions of environmental changes^16^. Sensory impairment may endanger older adults’ health status, safety, activities of daily living, quality of life, and other factors contributing to life maintenance throughout the aging process^17,18^. Vision impairment has been associated with high levels of depression and anxiety and a risk of falls^17^. In addition, individuals with impaired vision may experience difficulties in engaging in activities of daily living, such as social and religious activities^17^^.^ Hearing impairment can reduce quality of life due to social isolation and physical dysfunction^18,19^. Individuals with sensory impairment were reported to have a higher risk of cognitive impairment than those without, and individuals with both vision and hearing impairment had a higher risk of cognitive impairment and dementia than those with no sensory impairment^20–22^.

Cognitive impairment, HGS, and sensory impairment are all included in the World Health Organization (WHO)'s proposed concept of intrinsic capacity for older adults﻿^23^. The WHO defined intrinsic capacity as the composite of all physical and mental abilities and functions that an individual can utilize throughout his or her lifetime^24^. Intrinsic capacity is a determinant of physical resilience and physiological reserve to withstand stressors and is an integrated indicator^25^. A comprehensive health assessment to monitor intrinsic capacity should include assessments of physical mobility and sensory, cognitive, vitality, and psychosocial functions of older adults^24^. In particular, HGS plays a substantial role in monitoring intrinsic capacity^26^. More evidence is needed to implement integrated and comprehensive healthcare plans drawing upon the results of monitoring intrinsic capacity in clinical and public health settings.

Nonetheless, it is difficult to determine the causal relationships between cognitive and sensory impairment through cross-sectional studies^21^. Although two large-scale studies have been conducted, their findings were limited in terms of understanding cognitive impairment and its relationship with sensory impairment in older adults, as the proportion of elderly individuals was only 6.4% in one study^21^ and the average age was 57.8 years in the other study^22^. Another longitudinal study analyzed the relationship between HGS and cognitive function, but only half of the subjects had HGS measurements every 2 years. Because the other half of the values were imputed, the results were likely to reflect overinference^5^. Concurrent lower HGS and sensory impairment are common in older adults^27^. Previous studies have analyzed the independent effects of either HGS or sensory impairment on cognitive impairment^5,10,14,28^. Thus, previous studies have not provided evidence regarding the combined effect of HGS and sensory impairment on cognitive impairment. Therefore, this study aimed to identify the association of the joint effects of both HGS and sensory impairment with cognitive impairment in older adults.

## Results

### Participant characteristics

This study included 2930 participants with a mean age of 73.5 years (standard deviation [SD] = 6.1), and 54.6% of the participants were women (Table 1). The participants’ mean score on the Korean version of the Mini-Mental State Examination (K-MMSE) was 24.9 points out of 30 (SD = 5.0), indicating normal cognitive function^29,30^. The normal-HGS group included higher proportions of individuals in the 65–75-year-old age group and men. The low-HGS group had a higher proportion of primary school graduates or less and a lower proportion of married participants. Furthermore, the low-HGS group had a high rate of physical inactivity (approximately 70%) and a high rate of never drinking alcohol (see Supplementary Table S1 online). Among the participants with dual sensory impairment, the proportion of those with multiple comorbidities was higher among those with low HGS than among those with normal HGS. Depressive symptoms were higher in the low-HGS group than in the normal-HGS group. The mean K-MMSE score was lowest in those with low HGS and dual sensory impairment (18.3 points; SD = 6.2).

**Table 1:** Demographic characteristics of the participants stratified by handgrip strength status and type of sensory impairment at baseline.

Variable	Total (n = 2930)	Normal handgrip strength (n = 1656)				*p*	Low handgrip strength (n = 1274)				*p*
		NI (n = 1263)	VI (n = 290)	HI (n = 73)	DI (n = 30)		NI (n = 766)	VI (n = 316)	HI (n = 79)	DI (n = 113)	
Age, mean (SD)	73.5 ± 6.1	71.4 ± 5.2	72.2 ± 5.2	73.9 ± 5.5	76.0 ± 5.3	***	74.5 ± 6.0	76.3 ± 6.1	78.7 ± 6.0	80.2 ± 6.3	***
Female, n (%)	1599 (54.6%)	650 (51.5%)	163 (56.2%)	27 (37.0%)	13 (43.3%)	*	411 (53.7%)	229 (72.5%)	45 (57.0%)	61 (54.0%)	***
Primary school or less, n (%)	1671 (57.0%)	595 (47.1%)	173 (59.7%)	34 (46.6%)	17 (56.7%)	**	454 (59.3%)	249 (78.8%)	59 (74.7%)	90 (79.6%)	***
Married, n (%)	2116 (72.2%)	990 (78.4%)	210 (72.4%)	61 (83.6%)	20 (66.7%)	*	544 (71.0%)	173 (54.7%)	56 (70.9%)	62 (54.9%)	***
K-MMSE, mean (SD)	24.9 ± 5.0	26.6 ± 3.6	24.6 ± 4.8	24.8 ± 4.1	23.3 ± 5.8	***	24.6 ± 4.8	22.3 ± 5.4	20.6 ± 6.7	18.3 ± 6.2	***

Analyzed using available data, including missing data. *P* value for the Pearson chi-square test for group comparisons of types of sensory impairment within each group of handgrip strength.

*DI* dual sensory impairment, *HI* hearing impairment only, *K-MMSE* Korean version of Mini-Mental State Examination, *NI* no sensory impairment, *SD* standard deviation, *VI* vision impairment only.

*p < 0.05, **p < 0.01, ***p < 0.001.

### The prevalence of cognitive impairment by follow-up period

**Figure 1:**
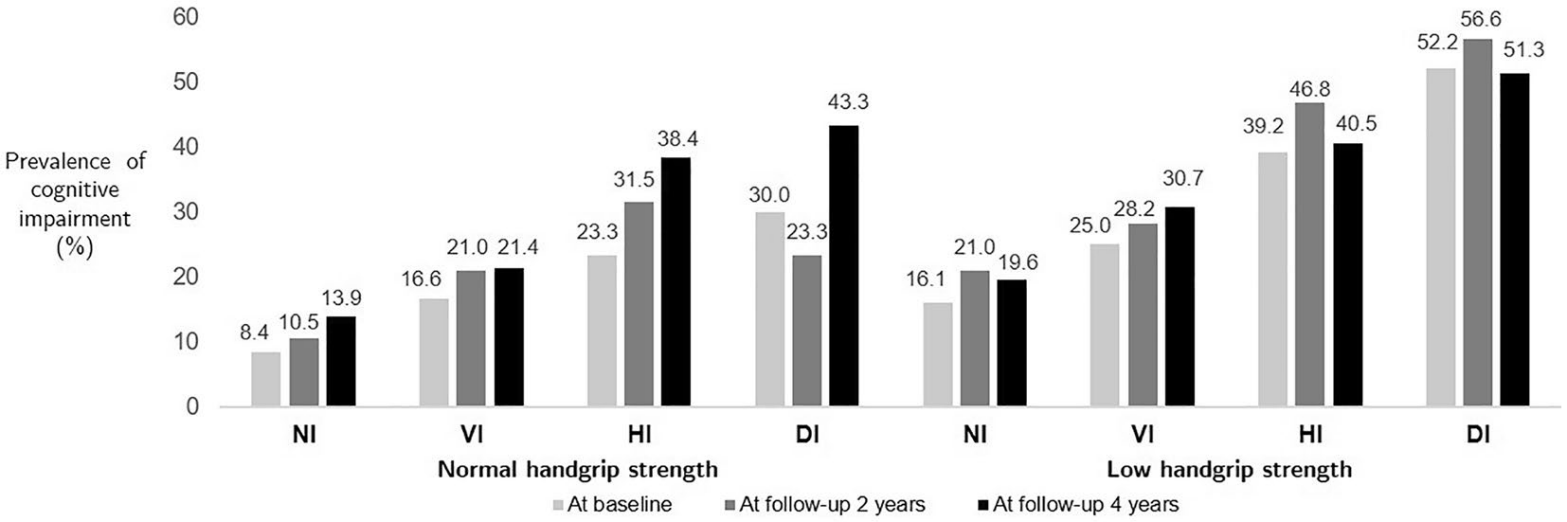
Prevalence of cognitive impairment stratified by handgrip strength status and type of sensory impairment by follow-up period. *DI* dual sensory impairment, *HI* hearing impairment only, *NI* no sensory impairment, *VI* vision impairment only.

An increasing trend in the prevalence of cognitive impairment was shown by the follow-up period when the participants were stratified by HGS status and type of sensory impairment (Table 2 and Fig. 1). The HGS‐stratified analysis showed significant differences across the types of sensory impairment at baseline, with a higher prevalence of cognitive impairment in participants with low HGS than in those with normal HGS: 16.1% versus 8.4% in those with no sensory impairment, 25.0% versus 16.6% in those with vision impairment, 39.2% versus 23.3% in those with hearing impairment, and 52.2% versus 30.0% in those with dual sensory impairment. The prevalence of cognitive impairment was higher in the participants with hearing impairment than in those with vision impairment in all HGS groups. The prevalence of cognitive impairment was highest in the participants with low HGS and dual sensory impairment at baseline, the 2-year follow-up, and the 4-year follow-up (52.2%, 56.6%, and 51.3%, respectively).

**Table 2:** The prevalence of cognitive impairment stratified by handgrip strength status and type of sensory impairment by follow-up period.

Period	Total (n = 2930)	Normal handgrip strength (n = 1656)				*p*	Low handgrip strength (n = 1274)				*p*
		NI (n = 1263)	VI (n = 290)	HI (n = 73)	DI (n = 30)		NI (n = 766)	VI (n = 316)	HI (n = 79)	DI (n = 113)	
At baseline, n (%)	472 (16.1%)	106 (8.4%)	48 (16.6%)	17 (23.3%)	9 (30.0%)	***	123 (16.1%)	79 (25.0%)	31 (39.2%)	59 (52.2%)	***
At follow-up 2 years, n (%)	575 (19.6%)	133 (10.5%)	61 (21.0%)	23 (31.5%)	7 (23.3%)	***	161 (21.0%)	89 (28.2%)	37 (46.8%)	64 (56.6%)	***
At follow-up 4 years, n (%)	615 (21.0%)	175 (13.9%)	62 (21.4%)	28 (38.4%)	13 (43.3%)	***	150 (19.6%)	97 (30.7%)	32 (40.5%)	58 (51.3%)	***

Analyzed using available data, including missing data. *P* value for the Pearson chi-square test for group comparisons of types of sensory impairment within each group of handgrip strength.

*DI* dual sensory impairment, *HI* hearing impairment only, *NI* no sensory impairment, *VI* vision impairment only.

*p < 0.05, **p < 0.01, ***p < 0.001.

### Combined effect of handgrip strength and sensory impairment on cognitive impairment

Binary logistic regression analyses using the generalized estimating equation (GEE) showed that in the normal-HGS group, those with vision, hearing, and dual sensory impairment (compared to those with no sensory impairment) had odds ratios (ORs) of 1.92, 3.46, and 3.64 for cognitive impairment, respectively (Table 3). The corresponding ORs for cognitive impairment in the low-HGS group were 1.36, 2.58, and 3.73, respectively. In the normal- and low-HGS groups, hearing impairment was associated with higher ORs for cognitive impairment (3.46 and 2.58, respectively) than were observed with vision impairment (1.92 and 1.36, respectively). The participants with dual sensory impairment also had a higher OR for cognitive impairment than those with single sensory impairment. The highest OR for cognitive impairment (3.73) was found in the participants with low HGS and dual sensory impairment.

**Table 3:** Adjusted odds ratios of sensory impairment for the prevalence of cognitive impairment according to handgrip strength status using generalized estimating equations.

Type of sensory impairment	Normal handgrip strength (n = 1656)		Low handgrip strength (n = 1274)	
	Adjusted OR (95% CI)	*p*	Adjusted OR (95% CI)	*p*
NI	1.00		1.00	
VI	1.92 (1.46, 2.53)	< 0.001	1.36 (1.06, 1.75)	0.016
HI	3.46 (2.33, 5.12)	< 0.001	2.58 (1.77, 3.78)	< 0.001
DI	3.64 (1.69, 7.84)	0.001	3.73 (2.65, 5.25)	< 0.001

Adjusted for age, sex, education level, marital status, physical activity, smoking, drinking alcohol, body mass index, number of comorbidity and depressive symptoms.

*CI* confidence interval, *DI* dual sensory impairment, *HI* hearing impairment only, *NI* no sensory impairment, *OR* odds ratio, *VI* vision impairment only.

**Figure 2:**
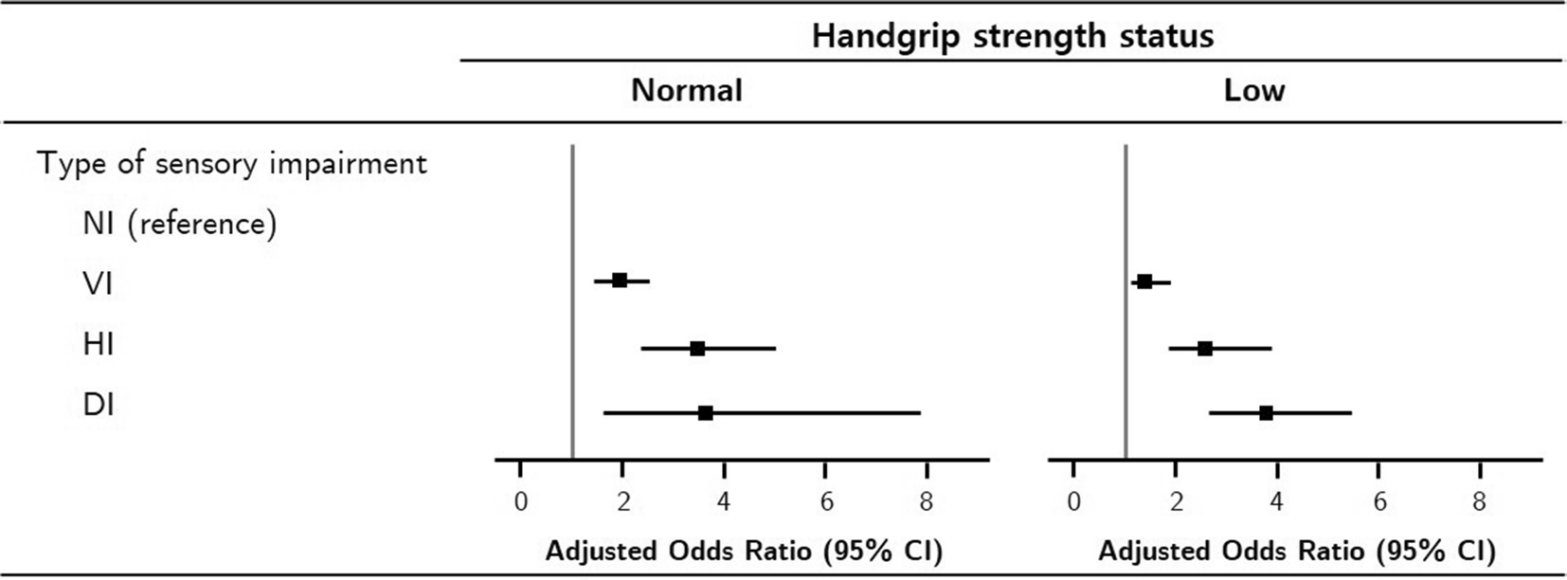
Forest plots of adjusted odds ratios of sensory impairment for the prevalence of cognitive impairment according to handgrip strength status. The adjusted odds ratios were shown as the squares, and the horizontal lines across the square represents for the 95% confidence intervals (CIs). Adjusted for age, gender, education level, marital status, physical activity, smoking, drinking alcohol, body mass index, number of comorbidity and depressive symptoms. *CI* confidence interval, *DI* dual sensory impairment, *HI* hearing impairment only, *NI* no sensory impairment, *VI* vision impairment only.

The adjusted ORs related to sensory impairment regarding the prevalence of cognitive impairment based on HGS status were further presented using forest plots (Fig. 2). Figure 2 clearly shows differences in the prevalence of cognitive impairment according to HGS status and type of sensory impairment, and the ORs showed a similar trend in the participants with normal HGS and low HGS. However, the participants with a combination of low HGS and dual sensory impairment had the highest OR for cognitive impairment; this combination was significantly associated with an increased risk of cognitive impairment.

## Discussion

In this large representative sample of older adults, this study found a combined effect of HGS and sensory impairment on cognitive impairment. Stratified associations by HGS showed a higher prevalence of cognitive impairment in the participants with low HGS with sensory impairment than in those with normal HGS. Hearing impairment was more associated with cognitive impairment than vision impairment. And dual sensory impairment was more strongly associated with cognitive impairment than single sensory impairment. Cognitive impairment was highest in the participants with low HGS and dual sensory impairment.

Compared to normal HGS, low HGS was associated with old age, female, and low educational level. The rate of physical inactivity was high in the low-HGS group. In research results from the United Kingdom, Sweden, and Denmark, participants with characteristics such as old age, female sex, and physical inactivity also consistently showed low HGS^9,10,31^. This study is meaningful in that it provides health information on the characteristics of older adults in Asia who are experiencing rapid aging based on their HGS and sensory impairment. By doing so, these results enhance researchers’ understanding of the elderly population. In addition, the follow-up period of 4 years in this study was from 2014 to 2018; in 2018, older adults accounted for 14.3% of the total population of Korea, which has become an aged society^32^. These findings serve as a meaningful basis for health promotion projects and healthcare policies for older adults in countries that are about to become aged or super-aged societies.

The stratified associations by HGS showed a higher prevalence of cognitive impairment in the low-HGS group than in the normal-HGS group. Several studies have shown that low HGS is associated with cognitive function and cognitive impairment^4,5,13^. A previous study reported a relationship between HGS and brain atrophy using brain magnetic resonance imaging (MRI) in individuals aged 70 to 73 in the United Kingdom^33^. Muscle function reflects aging in specific regions of the brain (white matter lesions) rather than the whole brain^33,34^. Muscle strengthening exercises and physical activity can help reduce the decrease in the number of nerve cells in the brain and maintain the synaptic plasticity of nerve cells^35^. Therefore, it would be meaningful to apply an intervention program to reduce aging-related cognitive impairment, and there is a rationale for including HGS as an indicator. However, previous studies of interventions involving cognitive training, exercise, and vitamin supplementation have shown little or no effect on the prevention or delay of cognitive impairment^36^. Of note, cognitive training was reported to improve cognitive function at 2 years but not at 5 years. With a long follow-up period, attrition may occur due to health problems (comorbidities) or death^36^. Future studies should be aware of issues such as the follow-up period, attrition, and intervention dosage (minutes per session and total duration) and should be designed as randomized controlled trials.

In both the normal- and low-HGS groups, vision impairment had a significant relationship with cognitive impairment. Some studies have shown a significant relationship between vision impairment and cognitive impairment^21,22,37^, but the opposite results have also been reported^38,39^. No clear difference was found according to the characteristics of the study participants (age, sex, and education level), country, study design, and measurement of vision impairment (i.e., through objective measurements or subjective self-reports). A longitudinal study of the association of vision impairment with cognitive impairment in various settings and populations should be conducted.

The risk of cognitive impairment was higher in those with hearing impairment than in those with vision impairment in both the normal- and low-HGS groups. The Canadian Longitudinal Study on Aging, which investigated the relationship between objectively measured sensory impairment and cognitive decline, reported that hearing impairment had a more negative effect than vision impairment on executive function and memory in cognitive domains, which is consistent with our findings^40^. Our results support previous studies by reporting that hearing impairment was significantly associated with cognitive impairment and risk for dementia in research with a longitudinal design^20,39,41^. Lin et al.^42^ found that the risk of cognitive impairment increased as the level of hearing impairment increased among older adults in the United States. Hearing impairment causes communication problems in forming relationships, interacting with others, and engaging in social activities, loneliness, social isolation, and poor quality of life^18,19^. Hearing impairment requires more cognitive resources for auditory perceptual processing due to the impoverishment of auditory signals when communicating. Furthermore, other cognitive tasks, such as working memory, are also mobilized for effortful listening with attention and concentration, which depletes one’s cognitive reserve. The cognitive load in auditory perceptual processing causes brain structural changes and neurodegeneration^43–45^. In particular, hearing loss causes changes in the volume of the primary auditory cortex and the total brain^46,47^. Although the causal relationship between hearing impairment and cognitive impairment remains to be elucidated, hearing impairment is a risk factor that can be modified as a preventive dimension for cognitive impairment^41^. Healthcare providers should evaluate aging-related hearing problems and the physical, psychological, and social aspects of negative outcomes. Appropriate management is also needed according to the severity of hearing impairment. For example, mild hearing impairment necessitates regularly monitoring hearing function, controlling environmental risk factors, and encouraging meaningful communications with intimate others. A hearing aid is useful for severe hearing problems. Hearing aid use has been reported to result in cortical changes and neurocognitive compensation^41^. Further research is needed to identify mediators (e.g., communication, depression, and loneliness) that accelerate or delay cognitive impairment associated with hearing impairment.

In both the normal- and low-HGS groups, the presence of dual sensory impairment was associated with a higher risk of cognitive impairment than the presence of single sensory impairment. This is consistent with the results of previous studies reporting that dual sensory impairment increase the risk of cognitive decline and cognitive impairment^22,48^. Multiple studies of sensory impairment have found that dementia increases concomitantly with the number of sensory impairments^39^. These results can serve as a key indicator for detecting and predicting cognitive impairment at an early stage by measuring the sensory function of older adults with normal HGS as a reflection of their physical function. Cognitive impairment has a significant negative effect on healthy cognitive aging in older adults, and sensory impairment can be used to predict potential cognitive impairment through an accurate, easy, and quick measurement of sensory function. A standardized diagnostic test of sensory function that can evaluate cognitive impairment and research on its sensitivity and specificity are needed.

The prevalence of cognitive impairment was highest in the participants with low HGS and dual sensory impairment. Previous studies have identified HGS or sensory impairment to be independent factors in cognitive impairment^5,10,22,41^. The present results reflect the combined effect of HGS and sensory impairment on cognitive impairment among older adults. Monitoring intrinsic capacity for physical mobility, sensory, cognitive, vitality, and psychosocial functions^24^ includes assessments of HGS, sensory impairment, and cognitive impairment, which are the main concepts investigated in this study. In other words, based on the results of monitoring intrinsic capacity, including HGS, sensory impairment, and cognitive impairment, older adults with low HGS and dual sensory impairment should be classified as a high-risk group with regard to cognitive impairment. These results provide a rationale for the optimal timing and components (HGS and sensory function) for cognitive stimulation interventions as part of an integrated and comprehensive healthcare plan to improve intrinsic capacity and self-management. The risk of cognitive impairment can be reduced or delayed by applying an optimally timed intervention to older adults who have normal HGS and no sensory impairment (i.e., before the onset of low HGS and sensory impairment). Further studies are needed to evaluate the development and effectiveness of multidimensional interventions that simultaneously include muscle strengthening and improvement of sensory function as components of cognitive stimulation.

There were several limitations to this study when interpreting our findings. There should be caution about the effect of selective attrition bias in our longitudinal sample. Among the 4226 eligible participants at baseline, those who had missing or rejected measurement responses and those who did not participate in follow-up were excluded from the final analysis (n = 1296). The large number of dropouts may have led to an underestimation of the effects of HGS and sensory impairment on cognitive impairment. In addition to vision and hearing impairment, damage to the sense of smell is closely related to safety issues such as gas leakage and food freshness, damage to taste is related to nutritional issues, and damage to touch is closely related to falls^16^. Future studies should comprehensively evaluate the effects of sensory impairment, including vision, hearing, smell, taste, and touch, on cognitive impairment. Despite these limitations, a strength of this study is that it investigated the combined effects of HGS and sensory impairment on cognitive impairment using longitudinal data at a 4-year follow-up.

Low HGS and sensory impairment were associated with the prevalence of cognitive impairment. The presence of dual sensory impairment was more strongly associated with cognitive impairment than the presence of single sensory impairment. This study makes a meaningful contribution through the novel finding that the combination of low HGS and dual sensory impairment is significantly associated with cognitive impairment. We recommend using HGS and sensory impairment as health indicators for the early identification of individuals with cognitive impairment and at-risk groups. Healthcare providers should monitor decreased HGS and changes in sensory function and provide interventions to prevent or delay cognitive impairment when it is reversible. Otherwise, the deterioration of cognition may accelerate, leading to irreversible cognitive impairment.

## Methods

### Study design

This study is a secondary data analysis of the Korean Longitudinal Study of Aging (KLoSA), which was conducted in 2014–2018.

### Setting and participants

The KLoSA dataset was collected for the purpose of understanding older adults’ actual situations in the process of the country transitioning to a super-aged society and establishing effective national policies^49^. The KLoSA was first conducted nationwide in 2006 and was followed up every two years until 2018. The KLoSA surveyed middle-aged and older adults (over the age of 45) who were comprehensively evaluated according to a range of parameters, including physical examination findings, health status, and socioeconomic status. Community-dwelling Korean adults were recruited from multiple locations and stratified by probability sampling^49^.

**Figure 3:**
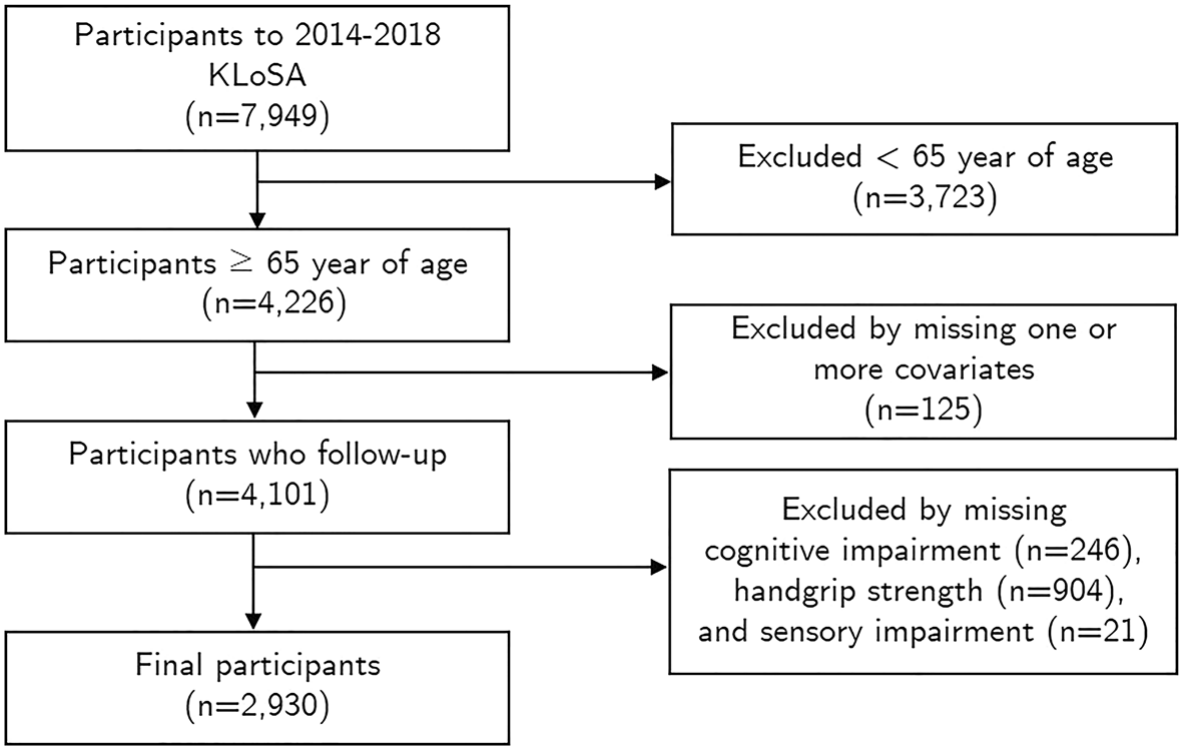
Flow of the selection process for this study sample. *KLoSA* Korean Longitudinal Study of Aging.

In the KLoSA, the samples were followed up for 12 years, with a sample retention rate of approximately 80%. In 2014, 920 people were added as a new sample, and survey items were added or changed in the demographic and health-related areas. For this reason, our study was conducted using the latest data from 2014 to 2018. The selection criteria for participants were those who (a) were aged 65 years and over, (b) participated in both the 2016 and 2018 follow-up surveys, (c) responded to all items measuring covariates, (d) had HGS and sensory impairment measurements as independent variables, and (e) had variables for cognitive impairment as the dependent variable for at least 2 out of the 3 measurements. For this study, a sample of 7949 adults was established in 2014, of whom 4226 people aged 65 years or older were selected. Due to loss to follow-up or responses that were missing or rejected, 1296 people were excluded. In total, 2930 participants were finally selected for study analysis (Fig. 3).

### Dependent variable: cognitive impairment

Cognitive impairment was measured using the K-MMSE scores from 2014 to 2018 KLoSA data. The scale consists of 19 items, including time/place orientation, short-term/long-term memory, language function, and executive function, with a total score range of 0 to 30^29,49^. Cognitive impairment was defined as a total K-MMSE score of less than 17 for respondents with no formal schooling, less than 20 points for those with a primary school education, and less than 24 points for those with a middle school or higher education^30^.

### Independent variables

#### Handgrip strength

HGS was measured in the right and left hands twice using a grip dynamometer (NO6103; Tanita, Tokyo, Japan), and the overall average was calculated^49^. According to the criteria of the Asian Working Group of Sarcopenia (AWGS), low HGS was defined as less than 28 kg for men and less than 18 kg for women^50^. In accordance with the AWGS criteria, the participants were categorized into the normal- or low-HGS group based on the baseline measurements.

#### Sensory impairment

Sensory impairment was defined based on responses to a questionnaire regarding vision and hearing. Sensory impairment was measured using a self-report questionnaire with a 5-point Likert scale, with 1 being ‘excellent’ and 5 being ‘poor.’ The participants were asked, “How is your eyesight?” This question further identified participants who generally used glasses or contact lenses. The participants also answered the question "How is your hearing?" This question also allowed the participants to specify whether they generally used hearing aids^49^.

Vision or hearing impairment was defined as a score of 4 or more in accordance with the criteria of Whillans and Nazroo^51^. The presence of dual sensory impairment (i.e., both vision and hearing impairment) was defined as a summed value of vision and hearing impairment of 8 or higher. The participants were classified according to the results of sensory impairment measured at baseline.

### Covariates

Several variables were included as potential confounders based on the literature^5,13,22^. Age, sex, education level, marital status, physical activity, smoking, drinking alcohol, BMI, the number of comorbidities, and depressive symptoms were investigated using 2014 KLoSA data. The participants were grouped as 65 to 74 years old or 75 years or older^52^. The highest level of education completed was divided into primary school or less, middle school, and high school or beyond. Marital status was classified as married or unmarried (bereaved, divorced, separated, or single). The participants were asked whether they engaged in physical activity more than once a week (yes or no). Smoking and drinking alcohol were each classified as never, former, and current. Based on the measured weight and height, ﻿BMI was calculated using units of kg/m^2^ and classified as follows: underweight (< 18.5), normal (18.5–22.9), overweight (23–24.9), and obese (> 25)^53^. Regarding comorbidities, the participants were asked if they had ever been diagnosed with hypertension, diabetes, cancer, chronic lung disease, cardiovascular disease, or cerebrovascular disease by a doctor. The total number of comorbidities was summed and classified as 0, 1, or ≥ 2. The Korean version of the 10-item short-form Center for Epidemiological Studies Depression scale (CES-D10) was used to evaluate depression^54^. The CES-D10 evaluates depressive symptoms experienced in the previous two weeks. Each question is rated on a 4-point scale ranging from 1 point for 'less than a day' and 4 points for '5–7 days', with higher scores indicating higher levels of depression.

### Ethical considerations

This study was conducted after obtaining approval for a screening exemption from the Institutional Review Board (no. Y-2020-0054) of Yonsei University in Seoul, Korea. The research was performed in accordance with the relevant guidelines and regulations. Informed consent was not sought for the research because the analysis utilized publicly available data.

### Statistical analysis

Raw data from the KLoSA from 2014 to 2018 were analyzed using SPSS 28.0 for Windows (IBM Corp., Armonk, NY, USA). For analyses stratified by HGS status and type of sensory impairment, numbers and proportions were given for categorical variables and means and standard deviations for continuous variables. The Pearson chi-square test was performed to determine the significance of differences in each variable according to the types of sensory impairment within each HGS group. To compare the prevalence of cognitive impairment, numbers and proportions were calculated by dividing HGS and sensory impairment according to the follow-up period. The dependent variable was analyzed binomially according to the presence of cognitive impairment. The effect of HGS and sensory impairment on cognitive impairment was assessed with a binary logistic regression model using GEEs. The binary logistic regression analyses were conducted with adjustments for age, sex, education level, marital status, physical activity, smoking, drinking alcohol, BMI, the number of comorbidities, and depressive symptoms. A GEE model is useful to probe how a dependent variable is correlated with the mean value for each time point in repeated measurements. It is characterized by statistical robustness since it reduces the errors arising from characteristics of the actual data that might differ from the population. Thus, the GEE method is optimal for maximizing data utilization and minimizing errors in parameter estimation in repeated-measurement data analysis^55^. A *p value* < 0.05 was considered to indicate statistical significance.

## Supplementary Information

Supplementary Information.
