# Supplementary material for: Combined effects of handgrip strength and sensory impairment on the prevalence of cognitive impairment among older adults in Korea
Source: Sci Rep. 2022 Apr 25;12:6713. doi: 10.1038/s41598-022-10635-9 (PMC9039062; doi:10.1038/s41598-022-10635-9)
Supplement: Supplementary file 1 — Supplementary Information. [file 41598_2022_10635_MOESM1_ESM.doc]

**Combined effects of handgrip strength and sensory impairment
on the prevalence of cognitive impairment among older adults in Korea**

**JuHee LEE**^1^**, Yujin SUH**^2^**, Jungah PARK**^3^**, Go-Un KIM**^4^**, Sumi LEE^5^**^＊^

^1^Mo-Im Kim Nursing Research Institute, Yonsei Evidence Based Nursing Centre of Korea: A JBI Affiliated Group, College of Nursing, Yonsei University, Seoul, 03722, Korea.

^2^College of Nursing, Health Science & Human Ecology, Dong-Eui University, Busan, 47227, Korea

^3^College of Nursing, CHA University, Pocheon, 11160, Korea

^4^College of Nursing, Inje University, Busan, 47392, Korea

^5^Department of Nursing, Graduate School, Yonsei University, Seoul, 03722, Korea

* Email: sumi022@naver.com

| **Variable** | **Total (n=2,930)** | **Normal handgrip strength (n=1,656)** | | | | ***p*** | **Low handgrip strength (n=1,274)** | | | | ***p*** |
| --- | --- | --- | --- | --- | --- | --- | --- | --- | --- | --- | --- |
|  |  | **NI (n=1,263)** | **VI (n=290)** | **HI (n=73)** | **DI (n=30)** |  | **NI (n=766)** | **VI (n=316)** | **HI (n=79)** | **DI (n=113)** |  |
| Physical activity |  | | | | |  |  | | | | *** |
| Yes, n (%) | 1041 (35.5%) | 485 (38.4%) | 102 (35.2%) | 27 (37.0%) | 13(43.3%) |  | 282 (36.8%) | 85 (26.9%) | 23(29.1%) | 24 (21.2%) |  |
| Smoking |  | | | | |  |  | | | |  |
| never, n (%) | 2025 (69.1%) | 848 (67.1%) | 192 (66.2%) | 42 (57.5%) | 17 (56.7%) |  | 542 (70.8%) | 247 (78.2%) | 58 (73.4%) | 79 (69.9%) |  |
| former, n (%) | 595 (20.3%) | 264 (20.9%) | 64 (22.1%) | 16 (21.9%) | 9 (30.0%) |  | 156 (20.4%) | 45 (14.2%) | 13 (16.5%) | 28 (24.8%) |  |
| current smoker, n (%) | 310 (10.6%) | 151 (12.0%) | 34 (11.7%) | 15 (20.6%) | 4 (13.3%) |  | 68 (8.8%) | 24 (7.6%) | 8 (10.1%) | 6 (5.3%) |  |
| Drinking |  | | | | |  |  | | | | *** |
| never, n (%) | 1563 (53.3%) | 639 (50.6%) | 155 (53.4%) | 31 (42.5%) | 15 (50.0%) |  | 403 (52.6%) | 204 (64.6%) | 43 (54.4%) | 73 (64.6%) |  |
| former, n (%) | 484 (16.5%) | 177 (14.0%) | 47 (16.2%) | 16 (21.9%) | 5 (16.7%) |  | 147 (19.2%) | 44 (13.9%) | 23 (29.1%) | 25 (22.1%) |  |
| current drinking  alcohol, n (%) | 883 (30.2%) | 447 (35.4%) | 88 (30.4%) | 26 (35.6%) | 10 (33.3%) |  | 216 (28.2%) | 68 (21.5%) | 13 (16.5%) | 15 (13.3%) |  |
| BMI, mean (SD) | 23.3±2.8 | 23.5±2.5 | 23.3±2.9 | 23.5±2.8 | 24.1±4.1 | ** | 22.9±3.0 | 23.0±3.1 | 22.4±3.3 | 22.2±2.8 |  |
| normal weight, n (%) | 1282 (43.8%) | 525 (41.6%) | 116 (40.0%) | 32 (43.8%) | 13 (43.3%) |  | 343 (44.8%) | 150 (47.5%) | 40 (50.6%) | 63 (55.8%) |  |
| underweight, n (%) | 128 (4.4%) | 21 (1.7%) | 13 (4.5%) | 0 (0.0%) | 2 (6.7%) |  | 52 (6.8%) | 22 (7.0%) | 10 (12.7%) | 8 (7.1%) |  |
| overweight, n (%) | 819 (28.0%) | 398 (31.5%) | 73 (25.2%) | 27 (37.0%) | 4 (13.3%) |  | 208 (27.2%) | 70 (22.2%) | 12 (15.2%) | 27 (23.9%) |  |
| obesity, n (%) | 701 (23.8%) | 319 (25.2%) | 88 (30.3%) | 14 (19.2%) | 11 (36.7%) |  | 163 (21.2%) | 74 (23.3%) | 17 (21.5%) | 15 (13.2%) |  |
| Number of comorbidity |  | | | | | *** |  | | | | *** |
| 0, n (%) | 1002 (34.2%) | 508 (40.2%) | 78 (26.9%) | 21 (28.8%) | 9 (30.0%) |  | 266 (34.7%) | 76 (24.1%) | 19 (24.1%) | 25 (22.1%) |  |
| 1, n (%) | 1104 (37.7%) | 452 (35.8%) | 123 (42.4%) | 23 (31.5%) | 13 (43.3%) |  | 288 (37.6%) | 135 (42.7%) | 33 (41.8%) | 37 (32.7%) |  |
| ≥ 2, n (%) | 824 (28.1%) | 303 (24.0%) | 89 (30.7%) | 29 (39.7%) | 8 (26.7%) |  | 212 (27.7%) | 105 (33.2%) | 27 (34.1%) | 51 (45.2%) |  |
| Depressive symptoms, mean (SD) | 1.9±1.9 | 1.7±1.8 | 2.1±1.7 | 2.0±2.0 | 2.3±2.2 | *** | 1.6±1.8 | 2.3±2.0 | 2.6±2.6 | 3.1±2.6 | *** |
| **Table S1.** Health-related characteristics of the participants stratified by handgrip strength status and type of sensory impairment at baseline. Analyzed using available data, including missing data. *P* value for the Pearson chi-square test for group comparisons of types of sensory impairment within each group of handgrip strength. BMI = body mass index; DI = dual sensory impairment; HI = hearing impairment only; NI = no sensory impairment; SD = standard deviation; VI = vision impairment only. **p*<.05, ***p*<.01, ****p*<.001. | | | | | | | | | | | |
